# Efficacy of Cefoperazone Sulbactam in Patients with *Acinetobacter* Infections: A Systematic Review of the Literature

**DOI:** 10.3390/antibiotics12030582

**Published:** 2023-03-15

**Authors:** Gowthami Sai Kogilathota Jagirdhar, Kaanthi Rama, Shiva Teja Reddy, Harsha Pattnaik, Rakhtan K. Qasba, Praveen Reddy Elmati, Rahul Kashyap, Marco Schito, Nitin Gupta

**Affiliations:** 1 Department of Medicine, Saint Michaels Medical Center, Newark, NJ 07104, USA; 2 Gandhi Medical College and Hospital, Secunderabad 500003, Telangana, India; 3 Lady Hardinge Medical College, New Delhi 110001, India; 4 Green Life Medical College and Hospital, Dhaka 1205, Bangladesh; 5 Interventional Pain Medicine, University of Louisville, Louisville, KY 40208, USA; 6 Critical Care Medicine, Department of Anesthesiology, Mayo Clinic, Rochester, MN 55092, USA; 7 CURE Drug Repurposing Collaboratory (CDRC), Critical Path Institute, 1730 E River Rd, Tucson, AZ 85718, USA; 8 Department of Infectious Disease, Kasturba Medical College, Manipal, Manipal Academy of Higher Education, Manipal 576104, India

**Keywords:** *Acinetobacter baumannii*, ventilator-associated pneumonia, antimicrobial resistance, multidrug resistance

## Abstract

Introduction: *Acinetobacter baumannii* (AB) is a multidrug-resistant pathogen commonly associated with nosocomial infections. The resistance profile and ability to produce biofilm make it a complicated organism to treat effectively. Cefoperazone sulbactam (CS) is commonly used to treat AB, but the associated data are scarce. Methods: We conducted a systematic review of articles downloaded from Cochrane, Embase, PubMed, Scopus, and Web of Science (through June 2022) to study the efficacy of CS in treating AB infections. Our review evaluated patients treated with CS alone and CS in combination with other antibiotics separately. The following outcomes were studied: clinical cure, microbiological cure, and mortality from any cause. Results: We included 16 studies where CS was used for the treatment of AB infections. This included 11 studies where CS was used alone and 10 studies where CS was used in combination. The outcomes were similar in both groups. We found that the pooled clinical cure, microbiological cure, and mortality with CS alone for AB were 70%, 44%, and 20%, respectively. The pooled clinical cure, microbiological cure, and mortality when CS was used in combination with other antibiotics were 72%, 43%, and 21%, respectively. Conclusions: CS alone or in combination needs to be further explored for the treatment of AB infections. There is a need for randomized controlled trials with comparator drugs to evaluate the drug’s effectiveness.

## 1. Introduction

*Acinetobacter baumannii* (AB) is a multidrug-resistant (MDR) Gram-negative bacteria associated with nosocomial infections [1]. Its ability to cause nosocomial infections is partially due to its ability to survive on inanimate objects for a long duration of time [1]. In hospital settings, AB gets transmitted from these objects to patients through the healthcare worker’s hands [1]. It is commonly associated with outbreaks in critically ill hospitalized patients with hospital-acquired pneumonia and bloodstream infections as the most common presentations [1,2,3]. AB is becoming increasingly challenging to treat due to high rates of resistance to all antibiotics, including carbapenems [1,4,5,6,7,8]. Antibiotics such as polymyxin and tigecycline are commonly used to treat drug-resistant AB infections [1]. These agents are often expensive and are associated with myriad adverse events. Newer agents such as cefiderocol and eravacycline are unavailable in most resource-limited settings. Physicians worldwide, especially in low-resource settings, are forced to use antibiotics such as cefoperazone sulbactam (CS) with limited evidence for treatment in the absence of known effective therapies.

CS is a combination of beta-lactam (cefoperazone) and a beta-lactamase inhibitor (sulbactam). Cefoperazone is a broad-spectrum beta-lactam cephalosporin with activity against many Gram-negative bacteria [8]. Its activity is, however, compromised by the beta-lactamase produced by AB that cleaves the beta-lactam ring in this antibiotic. Sulbactam protects cefoperazone by destroying these beta-lactamase enzymes [8]. Additionally, sulbactam has intrinsic activity against AB, making this drug a candidate with suitable potential [8]. The drug is extensively used in resource-limited settings such as India. Still, the data on its effectiveness in this setting are scarce. This systematic review aims to evaluate the efficacy of CS alone or in combination therapy by calculating pooled clinical and microbiological cure rates and mortality for patients with AB infections.

## 2. Results

### 2.1. Inclusion of Studies

**Figure 1:**
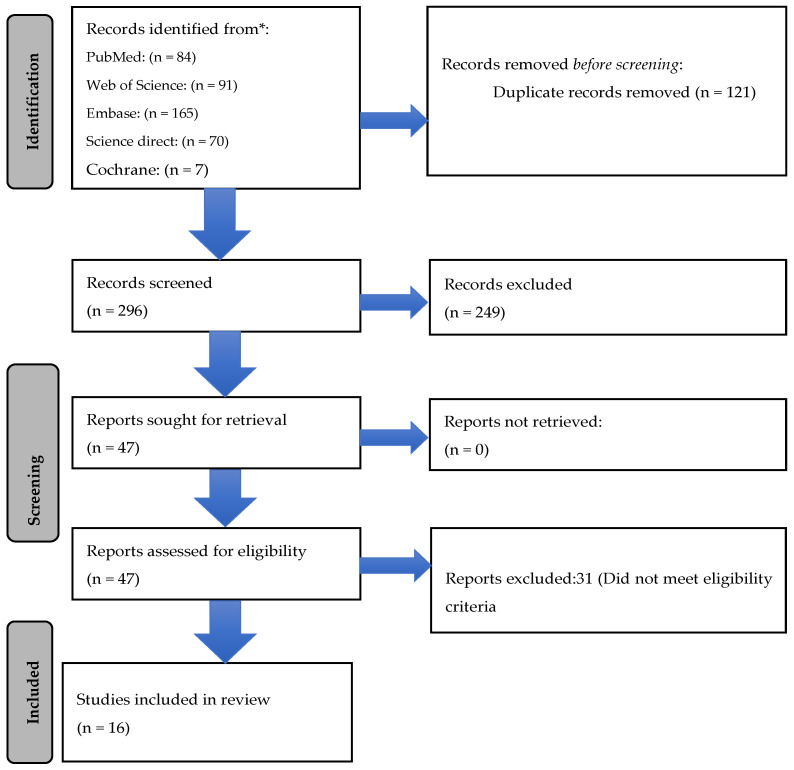
Prisma flow chart depicting studies including cefoperazone/sulbactam alone and in combination.

A total of 417 publications were identified from PubMed (n = 84), Web of Science (n = 91), Embase (n = 165), Science Direct (n = 70), and Cochrane (n = 7). After removing 121 duplicates, a total of 296 articles were screened by two independent reviewers (Figure 1). Of these, 47 articles were selected for full-text screening. After excluding 31 articles that did not meet the eligibility criteria, a total of 16 articles were finally included for data extraction and analysis [9,10,11,12,13,14,15,16,17,18,19,20,21,22,23,24].

We separated the 16 studies into 2 groups based on whether CS was used alone or in combination with other antibiotics (carbapenems, polymyxins, tigecycline/minocycline) to treat AB. Six studies reported patients that were only treated with CS alone. Five studies included patients where CS was used only in combination with antibiotics. In five studies, both types of patients where CS was used alone and in combination were included.

### 2.2. Assessment of Quality

The assessment of the quality of the studies using the NIH Quality Assessment Tool found the majority of the studies to be of fair or suitable quality.

### 2.3. Baseline Characteristics in Patients Where CS Was Used Alone

A total of 11 studies treating AB infections with CS alone were identified (Table 1). All the studies were observational, and no RCTs were found to meet our selection criteria. All the studies were from Asia (China (n = 7), Korea (n = 1), Taiwan (n = 1), and Thailand (n = 2)). The most common infections encountered when CS was used alone were pulmonary infections (n = 191) followed by intra-abdominal infections (n = 18), bloodstream infections (n = 16), skin and soft tissue infections (n = 8), and urinary tract infections (n = 2).

### 2.4. Baseline Characteristics When CS Was Used in Combination

A total of 10 studies were identified in which AB infections were treated with CS in combination with other antimicrobial agents (Table 2). Except for one randomized controlled trial, all were observational studies or case series. Except for one report from Turkey, all the reports were from Asia (China (n = 8), Thailand (n = 1)). The other antimicrobial agents used in combination with CS were colistin, tetracyclines (doxy/minocycline), tigecycline, and carbapenems. The most common infection treated with CS in combination with other antimicrobials was pneumonia (n = 166), followed by bacteremia (n = 49), skin-soft tissue infection (n = 8), intra-abdominal infection (n = 7), and urinary tract infection (n = 7).

### 2.5. Antimicrobial Susceptibility, Dosage of CS, and Outcome of Patients When CS Was Used Alone

Of the 11 included studies, antimicrobial susceptibility was available in only 7 studies. Of these seven studies, all cases were carbapenem-resistant, except in one study where 89% of isolates were carbapenem sensitive [2]. Colistin and tigecycline susceptibility was known in only one study [18]. All the tested isolates in that study were sensitive to both tigecycline and colistin (Table 3). CS susceptibility was available in only two studies where it was shown to be 100% susceptible. CS was used alone in these 11 studies, but the dosage was mentioned in only 6 studies (Table 4). In the six studies where the dosages were mentioned, 3 g every 6 to 8 hours was used in all but one study. The duration of antibiotic duration ranged from 1 to 4 weeks (Table 4). Of the 153 patients with clinical outcome data, 107 (69.9%) had positive clinical outcomes. Of the 141 patients with microbiological outcome data, 65 (46%) patients showed culture negativity on repeat sampling (Table 4). Of the 198 patients with available mortality data, 39 (19.6%) died despite CS treatment (Table 4).

### 2.6. Outcome of Patients When CS Was Used in Combination

CS was used in combination with various antibiotics. Combination with tigecycline, carbapenems (with or without other antibiotics), and colistin were used in four, three, and two studies, respectively. Susceptibility reports were available for all 10 studies. All isolates of AB included in this group were carbapenem-resistant. Four studies included tigecycline-resistant cases, while the other two had tigecycline-sensitive isolates. (Table 5). All cases were susceptible to polymyxin. CS susceptibility was available in only three studies, and all the tested isolates were resistant. The dosage of CS was available for only six studies. The most common dosage for adults was 3 g every 6 to 12 hours. The most common duration of antibiotics was one to six weeks. Of the 71 cases in which clinical outcome data were available, the pooled clinical cure rate was 63.3% (45/71). Of the 122 patients where repeat microbiological cultures were available, 52 (42.6%) patients had demonstrated culture conversion (Table 4). The pooled mortality rate was 30.7% (20/65) (Table 6).

## 3. Discussion

Rising AMR has been a cause of urgent concern in recent times, with WHO rating it as one of the most significant public health threats [25]. Among the pathogens with the highest resistance rates, the ‘ESKAPE pathogens’ have the highest impact on outcomes [25]. AB is one of the ESKAPE pathogens with demonstrable resistance to all the commonly used antibiotics, including carbapenems [25]. Although complete susceptibility reports were not available in most studies included in the review, overall, the resistance rates were high. All the tested isolates for AB across the studies were mostly resistant to carbapenems. Polymyxin was found to be susceptible for all tested isolates, but it must be noted that the only recommended method for polymyxin susceptibility is the microbroth dilution method, which was not performed in any of the studies. In the absence of susceptibility reports determined by approved methods, it is difficult to draw conclusions. Tigecycline susceptibility was variable across the included studies. CS was 100% susceptible in two studies, while it was 100% resistant in three studies. It was given as monotherapy in those where it was susceptible but was given as combination therapy in resistant cases. In addition, it must be noted that the susceptibility of CS is not standardized, and evidence-informed cut-offs are often unavailable.

The correct dosage of CS and the ratio of cefoperazone and sulbactam has not been unanimously agreed upon. The commonly used dosage in our review for cefoperazone/sulbactam was 3 g every 6 to 12 h. Studies on CS’s pharmacokinetic/pharmacodynamic (PK/PD) index in AB infections are limited [26]. It is also unclear what the optimal ratio is to combine cefoperazone and sulbactam. Cefoperazone and sulbactam are usually combined in a ratio of 2:1. In vivo studies have suggested that a ratio of 1:1 or 1:2 might be better for multidrug-resistant infections such as carbapenem-resistant AB. [27]. Since sulbactam has intrinsic activity, it has been suggested that adjusting sulbactam dosage can increase the microbiological effects of the combination [28]. It has also been suggested that increasing the infusion time may also increase the effectiveness of the drug [26]. It must be noted that based on the elimination characteristics, dose adjustment may be required in severe biliary obstruction and hepatic or renal dysfunction [29].

According to the latest guidelines by the Infectious Disease Society of America (IDSA), there is no defined standard of care for AB infections, especially the ones with carbapenem-resistant phenotypes [30]. The IDSA guidelines suggest monotherapy for mild infections and combination therapy for moderate to severe infections [30]. The categorization of the severity of illness was not described in most of the included studies. The IDSA guidelines suggest ampicillin sulbactam as the treatment of choice for mild AB infections [30]. The limited data on the efficacy of ampicillin sulbactam suggest that it is one of the drugs most effective for treating AB infections [31,32,33,34,35,36,37,38]. These data can be partially extrapolated to other sulbactam-containing beta-lactam combinations such as CS. It must be noted that ampicillin sulbactam might not be available in resource-limited settings, and CS is not available in the United States of America. Therefore, the IDSA guidelines did not comment on the utility of CS. For severe infections, the guideline suggests combining high-dose ampicillin sulbactam with drugs such as colistin and tigecycline [30].

In this SR, CS was used as a single agent in some studies and in combination with other drugs in other studies. The use of CS as a standalone agent was associated with a considerable response in most cases. The pooled clinical and microbiological cure rates were 70% and 46%, respectively. Considering the lack of consensus for treatment and the poor quality of data for any single drug, combination therapy has been tried in several studies. Some authors also recommend the use of CS in combination because the standalone use of CS has been shown to result in quicker resistance development [39]. On the other hand, it must also be noted here that evidence favoring the use of combination therapy in AB is not uniform. Of the seven trials comparing mono vs. combination therapy for AB reported by IDSA, only one trial showed definite benefit with combination therapy [37,40,41,42,43,44,45].

CS, combined with tigecycline, colistin, and carbapenem, showed a considerable response in our SR. The clinical cure rates of CS in combination with colistin, tigecycline, and carbapenem were 92% (12/13), 51% (22/43), and 71% (10/14), respectively, whereas the microbiological cure rates were 75% (9/12), 35.79% (34/95), and 64.28% (9/14), respectively.

There were only two studies that evaluated the colistin combination. When the results of these two trials were pooled, it showed that colistin combination with CS had the best overall response. This also corroborates with an SR, which showed that adding sulbactam to colistin improved clinical success [46]. It must be noted here that although the included studies used colistin, polymyxin can be used interchangeably in routine clinical settings. International consensus guidelines recommend polymyxin over colistin in most clinical situations (except urinary tract infection) because of its better pK-pD profile [47]. The IDSA guideline suggests caution against the colistin/polymyxin-containing combination owing to the high nephrotoxicity rates [30].

Our review suggests that outcomes with the combination of tigecycline and CS are less than desirable. Since most patients were of nosocomial pneumonia, and considering the poor track record of tigecycline in these patients, these results can be explained to a certain extent. The United States of America’s Food and Drug Administration issued a black box warning for the use of tigecycline monotherapy in pneumonia cases [48]. However, subsequent studies showed that increasing the dose of tigecycline helps in improving outcomes [49]. In light of the present data, tigecycline therapy with CS should be avoided, or when used, high-dose tigecycline may be more suitable.

The combination of carbapenem and CS showed modest efficacy despite the poor susceptibility to carbapenem reported in most studies. These results could be due to synergistic activity between the two drugs [50]. In another study, patients with bloodstream infections due to AB strains individually resistant to sulbactam and carbapenem were still susceptible to the combination of CS and imipenem [9].

AB is a nosocomial pathogen affecting people who are critically ill with several devices in situ. AB is also known to form biofilms on these devices [51,52]. Therefore, it is often challenging to differentiate between colonization and actual infection. In addition, since most patients who acquire nosocomial infection are critically ill with primary disease, the chances of poor outcomes at baseline are already high. The attainment of therapeutic drug levels in critically ill patients is also erratic. In such a situation, evaluating the efficacy of a drug becomes all the more difficult. It must be noted here that there was very high heterogeneity between the studies. Since most of the studies were of poor quality and the sample size was small, it is difficult to draw firm conclusions. The lack of randomized controlled trials on combination therapy, the risk of adverse effects, and resistance development complicate using combination therapy for AB. There is an ongoing unpublished randomized controlled trial comparing ceftazidime sulbactam with CS in patients with nosocomial pneumonia due to CRAB (ChiCTR1900024825). There is a need for additional randomized clinical trials to evaluate the efficacy of these drugs more conclusively.

## 4. Materials and Methods

### 4.1. Search Strategy

This systematic review (SR) includes pertinent articles in the literature on community or hospital-acquired, microbiologically proven AB infections that were treated with CS alone or in combination with another antibiotic(s). The following search terms were used in various databases: Pubmed (“Acinetobacter Infections” [Mesh] AND (Cefoperazone)), Web of Science (AB = (Acinetobacter infection) AND AB = (cefoperazone) AND AB = (sulbactam)), Embase (Acinetobacter AND infection AND cefoperazone AND sulbactam), Science direct ((“Acinetobacter Infections”) AND (Cefoperazone) AND (“Sulbactam”)), and Cochrane (Acinetobacter AND cefoperazone AND sulbactam AND Infection). Only title and abstract were searched in Cochrane, Embase, and Web of Science.

### 4.2. Inclusion and Exclusion Criteria

Randomized controlled trials, cohort studies, case control studies, cross-sectional studies, case series, and case reports were considered in our systematic review. Abstract-only articles, editorials, author responses, theses, conference abstracts, and books were excluded from this systematic review. The inclusion criteria for the study were community or hospital-acquired infections (pulmonary, bloodstream, central nervous system, intra-abdominal, urinary tract, skin/soft tissue, and osteoarticular) that were culture-positive for AB and were treated with CS. Animal model studies, plant studies, and in vitro studies were excluded. Clinical or microbiological cures and mortality rates are the outcomes of the current study. The study protocol was registered in Prospero ID CRC42022315925, and PRISMA guidelines were followed.

### 4.3. Screening and Full-Text Review

Rayyan web software was used for the screening and full-text review. A minimum of two investigators each screened the included articles. A third investigator was consulted for any conflicts. Abstracts of all studies were retrieved to identify records that met the inclusion criteria. Two investigators reviewed the articles chosen for full-text screening to assess if they met the inclusion criteria.

### 4.4. Data Extraction

The data were extracted from the final list of included articles. The essential demographic characteristics were recorded for all the studies, including the title, year of publication, author, study design, total number of participants, and study site/country. The symptoms of infection caused by AB were recorded wherever available. The patients were broadly categorized into pneumonia, bloodstream infection, urinary tract infection, skin-soft tissue infection, and intra-abdominal infection. Infective endocarditis was included within the purview of bloodstream infection. The categorization was performed according to the definitions used in the primary articles. The syndromes were not re-classified as community-acquired or nosocomial because it was assumed that the infections caused by AB would be primarily nosocomial. The antibiotic susceptibility pattern of carbapenem, tigecycline, and colistin was recorded for all the studies wherever available. An antibiotic was deemed susceptible or resistant according to the criteria used by the study. No additional cut-offs were applied to re-determine their susceptibility. The dose and duration of CS were recorded when used alone and when used in combination. When CS was used in combination, the details of the combination drug were also recorded. The duration of treatment in days was recorded for all patients. Three major outcomes were recorded wherever available: mortality rate, treatment failure, clinical cure rate, and microbiological cure rate.

### 4.5. Critical Appraisal of the Included Studies

For the appraisal of study quality, the NIH study quality assessment was used in case control, cohort, randomized controlled trials, and case series studies [53]. Each study was independently rated for quality by two reviewers using the appropriate tool based on the study design. As per the scale, studies were classified into three categories: good, fair, or poor, with 0 for poor (0–4 out of 14 questions), 1 for fair (5–10 out of 14 questions), and 2 for good (11–14 out of 14 questions). In addition, two authors independently performed the quality assessment for each included study. Finally, both authors discussed the article to reach a consensus if their ratings for the studies differed.

## 5. Conclusions

The limited data available in the literature suggested modest clinical outcomes with CS in patients with AB. Heterogeneity was ubiquitous, with diverse and complex patient profiles identified. Combination treatment, especially with polymyxins, can be tried for salvage therapy. There is a need for randomized controlled trials exploring the efficacy of CS with or without polymyxins for AB infections.

## Figures and Tables

**Table 1:** Types of infection in which cefoperazone-sulbactam was used alone.

	Author	Year	Type of Study	Pneumonia	Bloodstream Infection	Urinary Tract Infection	Skin-Soft Tissue Infection	Intra-Abdominal Infection
1	Chen et al. [9]	2015	Case Report		1 (100%)			
2	Choi et al. [10]	2006	Retrospective Observational Study	15 (43%)	13 (37%)	2 (6%)	1 (3%)	4 (11%)
3	Li and Wang et al. [11]	2017	Retrospective Observational Study	13 (100%)				
4	Li and Guo et al. [12]	2015	Retrospective Observational Study	4 (100%)				
5	Li and Xie et al. [13]	2020	Retrospective Observational Study	50 (71%)			7 (10%)	13 (19%)
6	Lin et al. [14]	2018	Case Report	1 (100%)				
7	Nakwan et al. [15]	2012	Retrospective Observational Study	3 (100%)				
8	Niu et al. [16]	2019	Retrospective Observational Study	7 (70%)	2 (20%)			1 (10%)
9	Pan et al. [17]	2016	Retrospective Observational Study	15 (100%)				
10	Yu et al. [18]	2021	Retrospective Observational Study	14 (100%)				
11	Wang et al. [19]	2022	Retrospective Observational Study	69 (100%)				

**Table 2:** Types of infection in which cefoperazone-sulbactam was used in combination.

	Author	Year	Type of Study	Pneumonia	Bloodstream Infection	Urinary Tract Infection	Skin-Soft Tissue Infection	Intra-Abdominal Infection
1	Arslan Gülen et al. [20]	2021	Retrospective Observational Study	7 (58%)	3 (25%)	1 (8%)	1 (8%)	
2	Li and Guo et al. [12]	2015	Retrospective Observational Study					
3	Li and Xie et al. [13]	2020	Retrospective Observational Study	50 (71%)		6 (9%)	7 (10%)	7 (10%)
4	Ning et al. [21]	2014	Retrospective Observational Study	7 (100%)				
5	Niu et al. [16]	2019	Retrospective Observational Study		45 (100%)			
6	Qin et al. [22]	2018	Randomized clinical trial	21 (100%)				
7	Xue et al. [23]	2021	Case report	1 (100%)				
8	Yu et al. [18]	2021	Retrospective Observational Study	28 (100%)				
9	Zhu et al. [24]	2019	Case report		1 (100%)			
10	Wang et al. [19]	2022	Retrospective Observational Study	52 (100%)				

**Table 3:** Method of antimicrobial susceptibility in studies where cefoperazone-sulbactam was used alone.

	Author	Method of Antimicrobial Susceptibility Testing				Percentage of Resistant Isolates			
		Carbapenem	Tigecycline	Polymyxin	CS	Carbapenem	Tigecycline	Polymyxin	CS
1	Chen et al. [9]	-	-	-	-	100			0
2	Choi et al. [10]	Disk diffusion	-	-	Disk diffusion	11			0
3	Nakwan et al. [15]	Disk diffusion	-	-	Disk diffusion	100			
4	Niu et al. [16]	-	-	-	-	100			
5	Pan et al. [17]					100			
6	Yu et al. [18]	VITEK 2 (bioMérieux)	VITEK 2 (bioMérieux)	VITEK 2 (bioMérieux)		100	0	0	
**7**	Wang et al. [19]	Agar dilution method				100			

**Table 4:** Outcomes of patients in whom cefoperazone-sulbactam was used alone.

	Author	Total Patients	Dosage of CS Per Day	Duration of Antibiotic (Days)	Outcomes		
					Clinical Cure % (N)	Microbiological Cure % (N)	All-Cause Mortality: N (%)
1	Chen et al. [9]	1		28	100 (1)	100 (1)	-
2	Choi et al. [10]	35			77.1 (27)	-	20 (7)
3	Li and Wang et al. [11]	35	9–12 g	-	71.4 (25)		5.7 (2)
4	Li and Guo et al. [12]	4	6–12 g	5–21	-	75 (3)	-
5	Li and Xie et al. [13]	66	9–12 g		70 (46)	50 (33)	5 (3)
6	Lin et al. [14]	1			100 (1)	100 (1)	-
7	Nakwan et al. [15]	3		7–14	-	-	33 (1)
8	Niu et al. [16]	30	3–8 g		-	-	40 (12)
9	Pan et al. [17]	15	9 g		45 (7)	-	26.6 (4)
10	Yu et al. [18]	69	9–12 g	8 (IQR 5–12.5)	-	39 (27)	-
11	Wang et al. [19]	14			-	-	71.4 (10)

**Table 5:** Methods of antibiotic susceptibility in studies where cefoperazone-sulbactam was used in combination with colistin or polymyxin, tigecycline, and carbapenem.

	Author	Method of Antimicrobial Susceptibility Testing				Percentage of Resistant Isolates			
		Carbapenem	Tigecycline	Polymyxin	CS	Carbapenem R%	Tigecycline R%	Polymyxin R%	CS R%
1	Arslan Gülen et al. [20]	Disc diffusion and VITEK 2.0 (bioMerieux)		Disc diffusion and VITEK 2.0 (bioMerieux)	-	100		0	
2	Li and Guo et al. [12]	Agar dilution		Agar dilution		100		0	
3	Li and Xie et al. [13]	Disc diffusion	Disc diffusion	-	Disc diffusion	100		0	
						100	0	0	
4	Ning et al. [21]	-	-	-	-	100	100	0	100
5	Niu et al. [16]	-	-	-	-	100		0	
6	Qin et al. [22]	-	Broth microdilution	-	Agar dilution method	100	0	0	100
7	Xue et al. [23]	-	-	-	-	100	100	0	100
8	Yu et al. [18]	Agar dilution method	Agar dilution method	Agar dilution method		100	0	0	
9	Zhu et al. [24]					100			
10	Wang et al. [19]		VITEK 2 (bioMérieux)	VITEK 2 (bioMérieux)			0	0	

**Table 6:** Outcome of patients treated with cefoperazone-sulbactam in combination with colistin or polymyxin, tigecycline, and carbapenem.

	Author	Total Patients	Combination Drug	Dosage of CS	Duration of Antibiotic	Clinical Cure % (n)	Microbiological Cure % (n)	Mortality % (n)
1	Arslan Gülen et al. [20]	12	Colistin			91.7 (11)	75 (9)	-
2	Li and Guo et al. [12]	4	Carbapenem			50 (2)	50 (2)	-
		3	Carbapenem and Tetracycline			33.3 (1)	33.3 (1)	-
3	Li and Xie et al. [13]	22	Tigecycline	3 g q8–12 h	>5 days	45 (10)	41 (9)	-
4	Ning et al. [21]	7	Carbapenem and Minocycline	12 g/day		100 (7)	88.89 (6)	-
5	Niu et al. [16]	35	Carbapenems	1–2 g q6–8 h		-	-	20 (7)
6	Qin et al. [22]	21	Tigecycline	Cefoperazone/3 g q6 h	14 days	85.71 (12)	9.52 (2)	-
7	Xue et al. [23]	1	Colistin	4.5 g q8 h	>7 days	100 (1)	-	0 (0)
8	Yu et al. [18]	28	Tigecycline			-	-	46 (13)
9	Zhu et al. [24]	1	Minocycline and carbapenem	3 g q6 h	42 days	100 (1)	0	0 (0)
10	Wang et al. [19]	52	Tigecycline	3 g q6–8 h	8 (IQR 5–12.5) days	-	44.2 (23)	-

## Data Availability

The authors confirm that the data supporting the findings of this study are available within the article.
